# Turning in Arm and Shoulder Presentations

**Published:** 1846-01

**Authors:** S. B. Phillips


*Turning, in Arm and Shoulder Presentations.* By S. B. Phillips, M.D.—Most works on obstetrics, and almost every professor, who lectures on this subject, give directions to seize both feet at the same time, in turning in utero, and bring them down together; or, if unable to grasp more than one, to secure that, after bringing it down, by the fillet; and then to introduce the hand again into the uterus, search for the other, and bring it through the external parts also, before attempting to proceed with the delivery.

This mode of procedure is so universally recommended and adopted, that almost every Obstetrician feels compelled to follow these general directions, lest he should become reprehensibly responsible, by departing from an established practice; but from my experience in turning, in arm and shoulder presentations, I am fully persuaded that it is far from being the *best* practice in all cases; but on the contrary, that it is pernicious and unnecessary in *most* instances, to re-enter the uterus after having obtained one extremity, for the purpose of grasping the other.

The operation is sufficiently eevere and dangerous, when performed with as little irritation as possible, without introducing the hand the second time; and the ease with which the delivery can be accomplished, by one extremity only, in all ordinary cases, conclusively proves that there is no necessity to re-enter the womb, and search for the other, thereby increasing the danger of rupturing the uterus, and producing inflammation of that organ.

Delivery by one extremity, has its advantages, which are not afforded by grasping both extremities at the same time; one of which, is the greater dilatation of the os uteri and external parts, in consequence of one extremity being folded upon the abdomen, consequently affording greater facility for the passage of the head, the only difficulty usually attending footling cases, and by far the most frequent cause of the death of the child, the funis umbilicalis being too long compressed.

I was *first* led to depart from the practice recommended by the high authorities in Midwifery, by the occurrence of the following case, which I will relate, as illustrative of the practice here recommended.

I was called about six o'clock, P.M., to Mrs. ——, in labor with her second child; she had arrived at full time. She informed me, that about one o'clock that morning, she was awakened by a "discharge of the waters," not being conscious of any pain; that for two or three hours prior to my arrival, she had experienced slight pains at distant intervals. Her pulse was 104, with high febrile excitement. On examination, I found the elbow at the os uteri, which was rigid and unyielding, dilated sufficiently to admit two fingers only, with difficulty.

I ascertained the position of the fœtus, through the abdominal parietes, with a good degree of certainty before entering the womb, though not always practicable; the head was felt in the left iliac region, the breech rather more elevated on the right side; and the feet, one or both, were more distinctly felt on the right of the median line, in the mesogastric region; by the last *distinctive* mark, I was enabled to distinguish the breech from the head, and that the *left* arm was presenting.

I exhibited one-eighth of a grain of antimonii et potassæ tartras, in solution, every fifteen minutes, which produced full emesis after the fifth dose, when I found the os uteri beginning to yield. In a few hours, it was sufficiently dilated to admit the hand freely, and without any improper force; whereupon, I entered the left hand, and seized the right foot, unable to obtain the other, at least, without much irritation. I brought it through the os externum, secured it by the fillet, and attempted to search for the other; but every trial to reintroduce the hand, (the liquor amnii having been discharged nearly twenty-four hours,) caused such pressing-down efforts on the part of the patient, without the occurrence of uterine contractions, that I twice relinquished the attempt, and concluded to deliver, if possible, by one extremity only. This I found more easy than I had anticipated. By making slight traction on the right extremity, the breech passed without the least difficulty, and the left extremity was at once liberated. The only delay in the case, was caused by the cervix uteri grasping the neck of the child, after the delivery of the arms;—therefore, I am constrained to believe, that had I delivered by both extremities, instead of one, I should have been compelled to use the forceps to deliver the head; the use of which, *ought always to be avoided if possible*, as well as the use of all other instruments, when delivery can be as well effected without them. She recovered without one untoward symptom, and as rapidly as in ordinary cases; but had I re-entered my hand, and repeated the cause which often produces rupture or inflammation, it is probable, that the effect would have been deleterious.

No rule can be laid down strictly applicable to all cases. It is not contended that delivery by one extremity is always preferable, but that it is generally the most judicious proceeding. On the contrary, in the case of a child escaping through a rent of the uterus into the abdominal cavity, I should consider the delivery by both extremities, the better mode, to avoid increasing the rupture already existing. In delivery by one extremity, it is important to seize that extremity which is opposite the presenting arm, or shoulder, else there may be some difficulty, in producing complete evolutions of the fœtus; and this may be more readily and completely produced by one extremity, than both, when that extremity is brought down, which is opposite to the presenting part; for by grasping an opposite extremity, a partial rotation of the body takes place, causing the presenting part to recede from the os uteri, as that extremity descends in the act of turning, thereby causing a more complete evolution of the fœtus, than if the presenting arm or shoulder remained at the os uteri.

Since the record of this case, I have seen a paper on turning, which recommends that evolution should be performed in cases of arm and shoulder presentations, by one extremity, and gives the preference to seizing the knee instead of the foot or ankle. This practice commends itself, inasmuch, as there would manifestly be less danger of rupturing the uterus, or of causing inflammation, which are frequently the consequences of the operation.

Whatever tends to render this operation less severe to the patient, and less dangerous in its consequences, is worthy of the candid, serious, and impartial consideration of the accoucheur, as it is an operation, imperiously demanded in many cases, for which no other means can be substituted, whereby it is possible to save the lives of both the mother and child.—*N. Y. Med. and Surg. Reporter.*

